# Association of High-Density Lipoprotein Subclasses with Chronic Kidney Disease Progression, Atherosclerosis, and Klotho

**DOI:** 10.1371/journal.pone.0166459

**Published:** 2016-11-18

**Authors:** Eiichiro Kanda, Masumi Ai, Mitsuyo Okazaki, Masayuki Yoshida, Yoshitaka Maeda

**Affiliations:** 1 Department of Nephrology, Tokyo Kyosai Hospital, Meguroku, Tokyo, Japan; 2 Department of Life Science and Bioethics, Graduate School of Medicine, Tokyo Medical and Dental University, Bunkyoku, Tokyo, Japan; 3 Department of Insured Medical Care Management, Graduate School of Medicine, Tokyo Medical and Dental University, Bunkyoku, Tokyo, Japan; 4 Tokyo Medical and Dental University, Bunkyoku, Tokyo, Japan; 5 Department of Nephrology, JA Toride Medical Center, Toride, Ibaraki, Japan; The University of Tokyo, JAPAN

## Abstract

**Background:**

Atherosclerosis is often a complication of chronic kidney disease (CKD) because of dyslipidemia and CKD-mineral and bone disorder. High-density lipoproteins (HDLs) are grouped into various subclasses composed of multiple proteins and lipids, and their transformation is altered in CKD. We investigated the roles of lipoprotein subclasses in CKD progression, and atherosclerosis, and the relationships with Klotho and fibroblast growth factor (FGF) 23.

**Methods:**

Seventy-one CKD patients were enrolled in this prospective cohort study in Japan. The proportions of cholesterol level to total cholesterol level (cholesterol proportion) and lipoprotein particle numbers in 20 lipoprotein fractions were measured by a newly developed high-performance gel permeation chromatography.

**Results:**

Diabetic nephropathy was observed in 23.9% of the patients. The mean age was 75.0 years and estimated glomerular filtration rate (eGFR) was 17.2 ml/min./1.73m^2^. The lipoprotein particle numbers in small HDLs were higher in Stage 4 group than in Stage 5 group (*p* = 0.002). Multivariate regression analysis adjusted for baseline characteristics showed that the cholesterol proportions in very small HDLs were associated with eGFR change rate [F19 *β* = -17.63, *p* = 0.036] and ABI [F19 *β* = 0.047, *p* = 0.047] in Stage 4 group, and that serum soluble α-Klotho level was associated with the lipoprotein particle numbers in very small HDLs [F19 *β* = 0.00026, *p* = 0.012; F20 *β* = 0.00041, *p* = 0.036] in Stage 5 group.

**Conclusions:**

This study showed that HDL subclasses are associated with CKD progression, ABI, and Klotho level in CKD-stage-specific manner.

## Introduction

Chronic kidney disease (CKD) patients have high risks of cardiovascular disease (CVD) [1, 2]. Atherosclerosis is often a complication of CKD because of dyslipidemia and CKD-mineral and bone disorder (CKD-MBD).

Lipid profile abnormalities vary depending on the urinary protein level and CKD stage. High levels of chylomicrons (CMs) and very-low-density lipoproteins (VLDLs) are observed [3, 4]. Although low-density lipoprotein cholesterol (LDL-C) levels are usually normal, small dense LDL-C levels are high and large LDL-C levels are low in CKD patients [5–7]. Moreover, high-density lipoprotein-C (HDL-C) levels are low. Nascent HDLs extract cholesterol from peripheral tissues, and transform into HDL3 with cholesterol ester storaged in its core. Cholesterol esterified by lecithin cholesterol acyltransferase (LCAT) is stored in the core of HDL3, and transforms HDL3 into HDL2.

CKD-MBD begins in the early CKD stage with a decrease in Klotho level and an increase in secreted fibroblast growth factor 23 (FGF23) level [8]. Interactions between FGF23, Klotho and lipid profile have been suggested. A cross-sectional study in elderly males showed that FGF23 is inversely associated with HDL-C level [9]. A dysfunctional variant of *KLOTHO* gene is inversely associated with HDL-C levels [10, 11].

HDL reduces CVD risk in the general population. However, HDL is not associated with lower mortality in CKD patients [12]. This suggests that not only cholesterol levels of lipoproteins but also the composition of lipoprotein subclasses may be one of the causes of the difference. To evaluate the roles of lipoproteins in CKD patients, measurements of not only lipoprotein cholesterol levels but also lipoprotein particle size are required. We previously established a method of high-performance gel permeation chromatography (HPGPC), with which we can separate lipoproteins into 20 fractions, and reported an association between lipid profiles and peripheral artery disease in peritoneal dialysis patients [13–15]. Therefore, the aims of this cohort study were to investigate (1) the lipid profiles at subclass levels in CKD patients, (2) the relationships between lipid profiles and CKD progression, and (3) the relationships between lipid profiles, ankle-brachial index (ABI) as a marker of atherosclerosis in the peripheral artery, and (4) levels of the CKD-MBD-related markers (Klotho and FGF23) using HPGPC.

## Materials and Methods

### Study design and study population

This is a prospective cohort study of CKD patients treated at the out-patient clinics of Tokyo Kyosai Hospital, Tokyo, Japan, and JA Toride Medical Center, Ibaraki, Japan. The patients were followed up for six months. The study was approved by the local ethics committees of Tokyo Kyosai Hospital, and JA Toride Medical Center. Written informed consent was obtained from each patient. We adhered to the evidence-based practice guideline 2013 for the treatment of CKD established by the Japanese Society of Nephrology [16]. Serum LDL-C level was maintained at less than 120 mg/dl by administration of statin. None of the patients was administered ezetimibe or fibrates. We excluded patients who had malignant diseases, infectious diseases, or severe liver diseases.

### Data

Patient demographics including age, gender, and history of diabetes mellitus (DM) as a cause CKD were obtained from the medical records of the patients at each hospital. ABI was calculated using the ratio of systolic blood pressure in the ankles to systolic blood pressure in the arms, which was derived from the mean of the right and left ratios. Blood samples were collected from every patient after overnight fasting. Routine serum biochemistry was carried out by standard methods at each hospital. Serum calcium level was adjusted for serum albumin level (4.0g/dl). Serum soluble α-Klotho (Klotho) and FGF23 levels were measured using an immunoassay kit (human soluble α-Klotho assay kit, Immuno-Biological Laboratories Co., Gunma, Japan; FGF-23 ELISA Kit, Kainosu laboratories Inc., Tokyo, Japan) at Skylight Biotech Inc, Akita, Japan. Lipoprotein fractions were analyzed by HPGPC as previously described [13–15]. HPGPC was carried out to simultaneously measure cholesterol levels and particle numbers in lipoprotein fractions. Cholesterol levels in lipoprotein fractions were analyzed by a mathematical procedure with modified Gaussian curve fitting to resolve overlapping peaks: CMs [Fraction (F) 1 and F2], large VLDLs (F3-F5), medium VLDL (F6), small VLDL (F7), large LDL F8, medium LDL F9, small LDL F10, very small LDLs (F11-F12), very large HDLs (F14-F15), large HDL F16, medium HDL F17, small HDL F18, very small HDLs (F19-F20). Considering the diameters of LDL particles, medium, small, and very small LDLs (F9—F13) are consistent with the classification of small dense LDL particles [13]. Very large HDLs (F14 and F15), large HDL (F16), and medium HDL (F17) correspond to HDL2, and small HDL (F18) and very small HDL (F19 and F20) to HDL3 [15]. The proportion of the cholesterol level in each lipoprotein fraction to total cholesterol level (cholesterol proportion) was calculated using the following formula: Cholesterol proportion (%) = cholesterol level in each lipoprotein fraction (mg/dl) / total cholesterol level (mg/dl) ×100.

Data on serum creatinine level were collected at baseline, and three and six months later. eGFR was calculated using the following equation for the Japanese population proposed by the Japanese Society of Nephrology [17]: eGFR (ml/min/1.73m^2^) = 194 × serum Cr^-1.094^ × age^-0.287^(for female) × 0.739, where Cr = serum creatinine level (mg/dl). eGFR decline (ml/min/1.73m^2^/month) was calculated using the least-squares method. Then, eGFR change rate was calculated: eGFR change rate (%/year) = eGFR decline/eGFR at baseline × 12. Negative level of eGFR change rate meant loss of kidney function. Rapid eGFR change was defined in this study as an eGFR change rate of less than -30%/year.

### Statistical analyses

Normally distributed variables are presented as mean ± standard deviation (SD); otherwise, the median and interquartile ranges are presented. Highly skewed variables were transformed with the natural logarithm function prior to their use in models [ln(urinary protein), ln(intact parathyroid hormone), ln(Klotho), ln(FGF23), ln(ABI)]. Intergroup comparisons were performed using the chi-square test, t-test, and Mann-Whitney U-test as appropriate. The differences in cholesterol proportions and lipoprotein particle numbers in lipoproteins between Stage 4 and 5 groups were evaluated by multivariate regression analysis adjusted for age, male, DM, urinary protein level, and statin use.

Univariate linear regression analysis was carried out to identify the factors which were associated with eGFR change rate (*p*<0.1). By CKD stage, multivariate linear regression analysis adjusted for the factors previously selected in the univariate linear regression analysis was carried out to determine whether the cholesterol proportion or particle number in each lipoprotein fraction was independently associated with eGFR change rate. Then, multivariate logistic regression analysis adjusted for the factors previously selected by univariate logistic regression analysis (*p*<0.1) was carried out to detect which cholesterol proportion or particle number in each lipoprotein fraction was independently associated with the rapid eGFR change by CKD stage. Similarly, the associations of cholesterol proportion level or particle number in each lipoprotein fraction with ln(ABI), ln(Klotho), and ln(FGF23) were examined by multivariate linear regression analysis by CKD stage. These analyses were conducted using SAS, version 9.4 (SAS, Inc., North Carolina, US). Statistical significance was defined as *p*<0.05.

## Results

### Patient characteristics and cholesterol levels in lipoproteins by CKD stage

Seventy-one patients were included as subjects for analysis. Of these, 36 patients were in CKD stage 4 (Stage 4 group), and thirty-five in CKD stage 5 (Stage 5 group). The patient demographics including biochemical data are shown in Table 1.

**Table 1:** Clinical and biochemical characteristics of patients in this study.

	All	Stage 4 group	Stage 5 group	*p*
N	71	36	35	
Age	75±11.1	76.9±9.6	73.1±12.4	0.16
Male (%)	50 (70.42)	29 (80.56)	21 (60)	0.058
Diabetes mellitus (%)	17 (23.94)	8 (22.22)	9 (25.71)	0.73
CVD (%)	24 (33.8)	13 (36.11)	11 (31.43)	0.68
BMI (kg/m^2^)	22.4±3.8	22.9±3.4	21.8±4.1	0.22
eGFR (ml/min/1.72m^2^)	17.2±8.3	23.9±5.9	10.2±2.8	0.0001*
Urinary protein (g/gcr)	1.9±2.5 0.99 (0.32, 2.29)	0.9±1 0.565 (0.205, 1.065)	2.9±3.1 1.65 (0.8, 3.98)	0.0001*
Adjusted calcium level (mg/dl)	9.3±0.7	9.5±0.5	9.1±0.7	0.0062*
Phosphorus (mg/dl)	3.9±0.9	3.4±0.6	4.5±0.9	0.0001*
Intact PTH (pg/ml)	247.7±269.1 162 (103.1, 257)	134.6±57.2 126 (99.85, 172.85)	364±343.9 220.2 (126.8, 518.6)	0.0004*
25-hydroxy vitamin D (ng/ml)	20.7±10.2	24±11.1	15.7±6.1	0.002*
FGF23 (pg/mL)	269.7±356.2 124.8 (81, 257)	98.7±60.2 89.45 (72, 113.2)	445.5±441.2 246.6 (165.7, 654.2)	0.0001*
Soluble α-Klotho (pg/mL)	509.4±147.6 494 (405.8, 584.1)	473.9±121.4 480.9 (386025, 535.7)	546±164.3 551.4 (432.2, 661.8)	0.053
ABI	1.1±0.1 1.09 (1, 1.14)	1.1±0.1 1.09 (1.03, 1.145)	1±0.2 1.07 (0.995, 1.14)	0.39
ARB ues (%)	47 (66.2)	22 (61.11)	25 (71.43)	0.36
Statin use (%)	25 (35.21)	17 (47.22)	8 (22.86)	0.032*
Precipitated calcium carbonate use (%)	5 (7.04)	0 (0)	5 (14.9)	0.019*
Vitamin D use (%)	22 (30.99)	11 (30.56)	11 (31.43)	0.94

Values are expressed as mean ± standard deviation. Urinary protein, intact PTH, FGF23, and soluble α-Klotho levels are expressed as median and interquartile range. The values are compared between the groups by the chi-square test, t-test and Mann-Whitney U test as appropriate.

*, *p*<0.05.

Abbreviations: CVD, cardiovascular disease; BMI, body mass index; eGFR, glomerular filtration rate; PTH, parathyroid hormone; FGF23, fibroblast growth factor 23; ABI, ankle-brachial index; ARB, angiotensin II receptor blocker.

**Fig 1:**
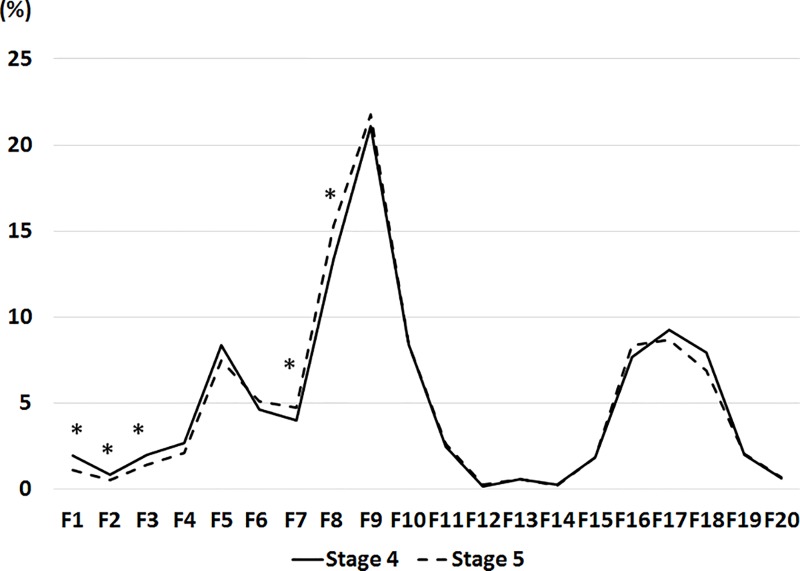
Cholesterol proportions in different lipoprotein fractions to total cholesterol level in Stage 4 group compared with those in Stage 5 group. The mean proportion of cholesterol level in each lipoprotein fraction to total cholesterol level is indicated in the graph. The values were compared between the groups by the t-test. *, *p*<0.05. Abbreviations: proportion, mean of the proportions of cholesterol level in each lipoprotein fraction to total cholesterol level; F1-20, Fractions 1–20.

The distributions of cholesterol and TG levels were examined (Table 2). No statistically significant difference in serum cholesterol levels in lipoproteins and serum TG levels between CKD stages were observed. The cholesterol proportion in each lipoprotein fraction is shown in Fig 1 and Table 3. In CM (F1 and 2) and large VLDL (F3), the cholesterol proportions in Stage 4 group were higher than those in Stage 5 group (Table 3, Fig 1). In small VLDL (F7) and large LDL (F8), the cholesterol proportions in Stage 4 group were lower than those in Stage 5 group. Although the difference in the cholesterol proportion in small HDL (F18) was not statistically significant, the cholesterol proportion was marginally higher in Stage 4 group than in Stage 5 group. After adjustment for age, male, DM, urinary protein level, and statin use, the differences in the cholesterol proportions between Stage 4 and 5 groups were examined. The differences in F2 and F3 between Stage 4 and 5 groups were statistically significant (F2, *p* = 0.029; F3, *p* = 0.008), and the difference in F1 was marginally significant (*p* = 0.079). However, the differences in F7, F8 and F18 were not significant (F7, *p* = 0.17; F8, *p* = 0.22; F18, *p* = 0.39).

**Table 2:** Cholesterol levels in lipoproteins and triglyceride levels.

	All	Stage 4 group	Stage 5 group	*p*
Total cholesterol (mg/dl)	169.9±36	176.9±39.8	162.7±30.6	0.096
VLDL-C (mg/dl)	36.8±14.9	39.5±18	34.1±10.4	0.13
LDL-C (mg/dl)	80.9±21.1	81.9±22.8	79.9±19.5	0.7
HDL-C (mg/dl)	48.4±15.3	50.6±16.5	46.2±13.9	0.23
Triglyceride (mg/dl)	146.3±91.5	162.2±86.3	129.9±94.9	0.14

Values are expressed as mean ± standard deviation. The values are compared between the groups by t-test. No significant diferences between cholesterol levels in lipoproteins and TG levels were observed between Stage 4 and 5 groups. Abbreviations: VLDL-C, very-low-density lipoprotein cholesterol; LDL-C, low-density lipoprotein cholesterol; HDL-C high-density lipoprotein cholesterol.

**Table 3:** Cholesterol proportions in lipoprotein fractions in Stage 4 group compared with those in Stage 5 group.

Class	Subclass	Fraction	All	Stage 4	Stage 5	*p*
CM		F1 (%)	1.51±1.72	1.92±1.84	1.1±1.51	0.044*
		F2 (%)	0.68±0.58	0.84±0.64	0.51±0.48	0.016*
VLDL	Large VLDL	F3 (%)	1.71±0.93	2±1.01	1.41±0.74	0.006*
		F4 (%)	2.41±1.3	2.69±1.42	2.11±1.11	0.061
		F5 (%)	7.93±2.45	8.38±2.82	7.47±1.94	0.12
	Medium VLDL	F6 (%)	4.85±1.73	4.62±1.97	5.09±1.43	0.26
	Small VLDL	F7 (%)	4.34±1.09	3.99±0.93	4.7±1.15	0.005*
LDL	Large LDL	F8 (%)	14.31±3.4	13.33±3.34	15.32±3.2	0.013*
	Medium LDL	F9 (%)	21.42±3.01	21.1±3.11	21.75±2.92	0.37
	Small LDL	F10 (%)	8.4±2.51	8.38±2.56	8.41±2.49	0.96
	Very small LDL	F11 (%)	2.54±0.76	2.46±0.68	2.61±0.83	0.42
		F12 (%)	0.21±0.2	0.17±0.17	0.25±0.21	0.094
		F13 (%)	0.56±0.13	0.57±0.15	0.55±0.11	0.66
HDL	Very large HDL	F14 (%)	0.24±0.19	0.26±0.24	0.22±0.13	0.37
		F15 (%)	1.83±1.36	1.82±1.45	1.84±1.29	0.94
	Large HDL	F16 (%)	8±5.83	7.67±6.84	8.34±4.64	0.63
	Medium HDL	F17 (%)	8.95±2.89	9.22±3.26	8.68±2.48	0.43
	Small HDL	F18 (%)	7.42±2.49	7.95±2.71	6.88±2.15	0.071
	Very small HDL	F19 (%)	2.01±0.55	1.97±0.57	2.05±0.53	0.58
		F20 (%)	0.67±0.15	0.65±0.13	0.69±0.17	0.21

*, *p*<0.05.

Abbreviations: CM, chylomicron; VLDL, very-low-density lipoprotein; LDL, low-density lipoprotein; HDL, high-density lipoprotein.

**Fig 2:**
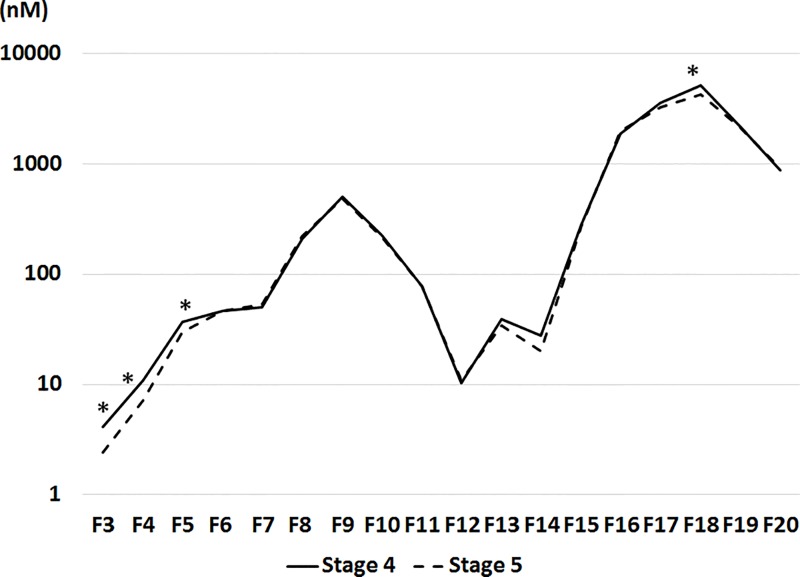
Lipoprotein particle numbers in different lipoprotein fractions in Stage 4 group compared with those in Stage 5 group. The mean proportion of cholesterol level in each lipoprotein fraction to total cholesterol level is indicated in the graph. The values were compared between the groups by the t-test. *, *p*<0.05. Abbreviations: proportion, mean of the proportions of cholesterol level in each lipoprotein fraction to total cholesterol level; F1-20, Fractions 1–20.

Lipoprotein particle numbers were also measured. In large VLDL (F3, F4 and F5), and small HDL (F18), the lipoprotein particle numbers were higher in Stage 4 group than in Stage 5 group (Table 4 and Fig 2). After adjustment for age, male, DM, urinary protein level, and statin use, these differences in the lipoprotein particle number between Stage 4 and 5 groups were statistically significant (F3, *p* = 0.0007; F4, *p* = 0.0016; F5, *p* = 0.0043; F18 *p* = 0.0073).

**Table 4:** Lipoprotein particle numbers in lipoprotein fractions in Stage 4 group compared with those in Stage 5 group.

Class	Subclass	Fraction	All	Stage 4	Stage 5	*p*
VLDL	Large VLDL	F3 (nM)	3.3±2.34	4.16±2.63	2.42±1.6	0.001*
		F4 (nM)	9.1±5.53	10.89±6.12	7.25±4.19	0.005*
		F5 (nM)	33.74±13.39	37.32±15.21	30.05±10.17	0.021*
	Medium VLDL	F6 (nM)	46.6±18.65	46.3±21.46	46.91±15.56	0.89
	Small VLDL	F7 (nM)	52.19±15.62	50.72±16.65	53.7±14.58	0.43
LDL	Large LDL	F8 (nM)	215.87±64.66	210.86±73.84	221.03±54.24	0.51
	Medium LDL	F9 (nM)	492.75±118.43	501.51±130.02	483.74±106.34	0.53
	Small LDL	F10 (nM)	223.37±75.93	229.01±77.24	217.56±75.25	0.53
	Very small LDL	F11 (nM)	77.62±26.07	77.91±24.03	77.32±28.36	0.92
		F12 (nM)	10.65±9.01	10.34±8.7	10.97±9.43	0.77
		F13 (nM)	36.62±12.16	38.9±14.34	34.28±9.03	0.11
HDL	Very large HDL	F14 (nM)	24.05±23.4	27.88±30.22	20.11±12.47	0.16
		F15 (nM)	274.86±201.45	278.66±208.91	270.95±196.45	0.87
	Large HDL	F16 (nM)	1934.1±1336.39	1878.26±1540.09	1991.53±1108.41	0.72
	Medium HDL	F17 (nM)	3444.14±878.92	3613.54±887.22	3269.89±847.64	0.1
	Small HDL	F18 (nM)	4733.74±1274.93	5194.57±1378.55	4259.76±966.5	0.002*
	Very small HDL	F19 (nM)	2190.36±498.55	2208.54±497.77	2171.66±505.92	0.76
		F20 (nM)	891.95±228.19	873.83±171.2	910.58±276.27	0.5

*, *p*<0.05.

Abbreviations: CM, chylomicron; VLDL, very-low-density lipoprotein; LDL, low-density lipoprotein; HDL, high-density lipoprotein.

### CKD progression

eGFR change rates are shown in Table 5. eGFR change rate was more rapid in Stage 5 group than Stage 4 group. The number of patients with a rapid eGFR change was larger in Stage 5 group than in Stage 4 group.

**Table 5:** CKD progression and CKD stage.

	All	Stage 4	Stage 5	*p*
eGFR change (%/year)	-23.3±30.5	-15.7±29.6	-31.0±29.9	0.034*
Rapid eGFR change (%)	18 (25.35)	4 (11.11)	14 (40.0)	0.0051*

*, *p*<0.05.

Abbreviations: eGFR, glomerular filtration rate; Rapid eGFR change, eGFR change less than -30%/year.

The relationship between cholesterol proportion in each lipoprotein fraction and eGFR change rate was examined. Univariate regression analysis showed that eGFR change rate was associated with ln(urinary protein) [parameter estimate (*β*) = -9.07 (standard error 2.71), *p* = 0.0014] and ARB use [*β* = 12.89 (7.56), *p* = 0.093]. In Stage 4 group, multivariate regression analysis adjusted for ln(urinary protein) and ARB use showed that eGFR change rate was negatively associated with cholesterol proportion in very small HDL (F19) (Table 6).

**Table 6:** eGFR change and cholesterol proportions and lipoprotein particle numbers in lipoprotein fractions.

	Cholesterol proportion				Lipoprotein particle number			
	Stage 4		Stage 5		Stage 4		Stage 5	
Fraction	Univariate	Multivariate	Univariate	Multivariate	Univariate	Multivariate	Univariate	Multivariate
F1	-4.09(2.68) 0.14	-4.25(2.51) 0.1	5.14(3.34) 0.13	1.49(4.26) 0.73				
F2	-7.32(7.87) 0.36	-8.45(7.42) 0.26	20.38(10.3) 0.056	14.19(13.19) 0.29				
F3	-2.46(5.01) 0.63	-2.99(4.79) 0.54	16.75(6.41) 0.013*	14.9(7.81) 0.066	-1.53(1.91) 0.43	-1.69(1.81) 0.36	4.65(3.15) 0.15	4.95(3.84) 0.21
F4	-1.44(3.57) 0.69	-1.49(3.45) 0.67	11(4.27) 0.015*	9.09(4.59) 0.057	-0.71(0.82) 0.4	-0.66(0.79) 0.41	1.6(1.21) 0.19	1.63(1.28) 0.21
F5	1.07(1.8) 0.56	1.36(1.73) 0.44	5.8(2.48) 0.026*	4.84(2.41) 0.055	-0.11(0.33) 0.75	0.014(0.34) 0.97	0.62(0.5) 0.22	0.6(0.46) 0.2
F6	1.12(2.58) 0.67	1.57(2.53) 0.54	2.2(3.62) 0.55	5.21(3.43) 0.14	-0.017(0.24) 0.94	0.051(0.24) 0.83	-0.15(0.33) 0.65	0.14(0.32) 0.67
F7	7.34(5.34) 0.18	7.17(5) 0.16	-6.75(4.38) 0.13	-1.59(4.7) 0.74	0.18(0.3) 0.55	0.25(0.29) 0.4	-0.78(0.33) 0.02*	-0.4(0.38) 0.3
F8	2.47(1.46) 0.1	2.66(1.4) 0.066	-2.34(1.57) 0.15	-0.4(1.76) 0.82	0.064(0.068) 0.35	0.081(0.063) 0.21	-0.21(0.089) 0.028*	-0.11(0.11) 0.31
F9	1.99(1.6) 0.22	1.9(1.56) 0.23	-1.61(1.76) 0.37	1.29(1.86) 0.49	0.029(0.039) 0.46	0.035(0.036) 0.34	-0.077(0.047) 0.11	-0.015(0.053) 0.78
F10	-1.46(1.97) 0.46	-1.54(1.87) 0.42	2.05(2.06) 0.33	2.66(2.06) 0.21	-0.025(0.066) 0.7	-0.022(0.063) 0.72	0.0089(0.069) 0.9	0.043(0.068) 0.53
F11	-9.4(7.27) 0.21	-7.9(6.96) 0.26	5.97(6.14) 0.34	6.97(6.24) 0.27	-0.18(0.21) 0.38	-0.13(0.2) 0.53	0.023(0.18) 0.9	0.088(0.18) 0.63
F12	6.53(29.99) 0.83	24.66(30.09) 0.42	31.05(23.59) 0.2	31.73(27.59) 0.26	-0.14(0.58) 0.81	0.14(0.59) 0.82	0.57(0.54) 0.3	0.56(0.65) 0.4
F13	-9.61(33.81) 0.78	13.57(33.25) 0.69	32.34(46.03) 0.49	-4.82(47.75) 0.92	-0.16(0.35) 0.66	0.072(0.35) 0.84	-0.15(0.58) 0.8	-0.44(0.6) 0.47
F14	0.29(21.45) 0.99	7.21(20.32) 0.72	25.13(40.03) 0.53	37.44(37.37) 0.32	-0.0092(0.17) 0.96	0.068(0.16) 0.68	0.25(0.41) 0.55	0.42(0.4) 0.3
F15	1.35(3.51) 0.7	1.4(3.31) 0.68	-2.25(4.03) 0.58	-5.77(3.89) 0.15	0.0069(0.024) 0.78	0.01(0.023) 0.66	-0.019(0.026) 0.47	-0.04(0.026) 0.1
F16	0.15(0.74) 0.84	0.06(0.7) 0.94	-0.94(1.11) 0.4	-1.72(1.05) 0.11	0.00044(0.0033) 0.89	0.00036(0.0031) 0.91	-0.0048(0.0046) 0.31	-0.0085(0.0044) 0.067
F17	0.15(0.74) 0.84	-1.33(1.56) 0.4	-1.82(2.07) 0.39	-3.09(2.02) 0.14	-0.00051(0.0057) 0.93	-0.0026(0.0059) 0.66	-0.0088(0.0059) 0.15	-0.011(0.0055) 0.063
F18	-3.17(1.8) 0.09	-2.63(1.74) 0.14	-0.71(2.42) 0.77	-2.15(2.64) 0.42	-0.0049(0.0036) 0.19	-0.0036(0.0035) 0.3	-0.0061(0.0053) 0.26	-0.0058(0.0052) 0.27
F19	-20.77(8.16) 0.016*	-17.63(8.04) 0.036*	0.63(9.84) 0.95	-6.82(9.49) 0.48	-0.022(0.0095) 0.025*	-0.019(0.01) 0.068	-0.0069(0.01) 0.5	-0.0075(0.0095) 0.43
F20	-80.91(36.75) 0.03*	-61.4(36.53) 0.1	11.46(29.97) 0.7	-31.73(29.86) 0.3	-0.0092(0.03) 0.76	0.011(0.029) 0.72	-0.013(0.019) 0.51	-0.024(0.02) 0.25

Relationships between eGFR change and serum lipid levels were evaluated by univariate or multivariate linear regression analysis adjusted for baseline characteristics. The values are expressed as parameter estimates (standard errors) and *p* values.

* *p*<0.05

Abbreviations: eGFR, glomerular filtration rate.

Then, the relationship between lipoprotein particle number and eGFR change rate was examined. In Stage 4 group, univariate regression analysis showed that lipoprotein particle number in very small HDL (F19) was negatively associated with eGFR change rate (Table 6). After the adjustment for ln(urinary protein) and ARB use, a marginal association was also observed in F19. In Stage 5 group, although eGFR change rate was negatively associated with lipoprotein particle numbers in small VLDL (F7) and large LDL (F8) in univariate regression analysis, these relationships were not statistically significant in multivariate regression analysis. The particle numbers in large HDL (F16) and medium HDL (F17) were found to be marginally associated with eGFR change rate.

A rapid eGFR change was associated with the cholesterol proportion in very small HDL in Stage 4 group: F19, odds ratio (OR) 7.102 [95% confidence interval (CI) 1.047, 48.163]; OR adjusted for ln(urinary protein) 7.813 (95% CI 1.056, 57.797). In Stage 5 group, a rapid eGFR change was not associated with cholesterol proportions or lipoprotein particle number.

### ABI and lipoproteins

Univariate regression analysis showed that ln(ABI) was associated with age [*β* = -0.00268 (0.00148), *p* = 0.076] and CVD [*β* = -0.08761 (0.03306), *p* = 0.010]. In univariate regression analysis, the relationship between ln(ABI) and cholesterol proportion was not observed both in Stage 4 and 5 groups (Table 7). However, multivariate regression models showed that, in Stage 4 group, ln(ABI) was positively associated with cholesterol proportion in very small HDL (F19). In Stage 5 group, ln(ABI) was negatively associated with cholesterol proportion in medium VLDL (F6).

**Table 7:** ABI and cholesterol proportions and lipoprotein particle numbers in lipoprotein fractions.

	Cholesterol proportion				Lipoprotein particle number			
	Stage 4		Stage 5		Stage 4		Stage 5	
Fraction	Univariate	Multivariate	Univariate	Multivariate	Univariate	Multivariate	Univariate	Multivariate
F1	0.0055(0.0073) 0.46	0.0056(0.007) 0.43	0.011(0.023) 0.63	0.0069(0.023) 0.77				
F2	0.017(0.0211) 0.41	0.0169(0.02) 0.41	0.0085(0.077) 0.91	-0.017(0.075) 0.82				
F3	0.0064(0.013) 0.63	0.0065(0.013) 0.62	-0.015(0.051) 0.77	-0.034(0.047) 0.47	0.0054(0.0051) 0.29	0.0052(0.0049) 0.3	-0.02(0.027) 0.46	-0.036(0.025) 0.16
F4	0.0069(0.0096) 0.48	0.0062(0.0093) 0.51	-0.026(0.033) 0.44	-0.044(0.03) 0.16	0.0025(0.0022) 0.26	0.0024(0.0021) 0.27	-0.0091(0.0094) 0.34	-0.015(0.0083) 0.08
F5	-0.0065(0.0047) 0.18	-0.0057(0.0046) 0.23	-0.018(0.017) 0.3	-0.02(0.017) 0.24	-0.00021(0.00093) 0.82	-0.00004(0.0009) 0.97	-0.0042(0.0033) 0.22	-0.0064(0.0031) 0.054
F6	0.0032(0.0077) 0.68	-0.00077(0.0078) 0.92	-0.038(0.023) 0.11	-0.046(0.02) 0.031*	0.00043(0.00072) 0.55	0.00023(0.0007) 0.74	-0.002(0.0021) 0.36	-0.0036(0.002) 0.08
F7	-0.0065(0.0147) 0.66	-0.0063(0.014) 0.66	-0.0075(0.029) 0.8	0.013(0.029) 0.66	0.000063(0.00083) 0.94	0.000094(0.0008) 0.91	-0.000027(0.0023) 0.99	0.00016(0.0023) 0.94
F8	-0.0033(0.004) 0.42	-0.003(0.0039) 0.45	-0.0073(0.011) 0.5	-0.00012(0.011) 0.99	-0.000068(0.00018) 0.71	-0.000039(0.00018) 0.83	-0.00029(0.0006) 0.64	-0.00029(0.00059) 0.63
F9	0.0024(0.0043) 0.59	0.0027(0.0042) 0.53	-0.0059(0.011) 0.6	-0.0056(0.01) 0.6	0.000045(0.0001) 0.67	0.000059(0.0001) 0.56	-0.00017(0.00029024) 0.57	-0.00032(0.00028) 0.25
F10	0.0068(0.0054) 0.21	0.0072(0.0051) 0.17	-0.0079(0.014) 0.59	-0.015(0.013) 0.25	0.00023(0.00018) 0.2	0.00025(0.00017) 0.16	-0.00033(0.00043) 0.46	-0.00065(0.00039) 0.11
F11	0.0258(0.021) 0.23	0.026(0.02) 0.22	-0.015(0.045) 0.75	-0.035(0.042) 0.41	0.00076(0.00059) 0.21	0.00077(0.00056) 0.18	-0.00063(0.0012) 0.6	-0.0014(0.0011) 0.2
F12	0.034(0.1) 0.74	0.046(0.1) 0.64	-0.15(0.2) 0.46	-0.21(0.18) 0.25	0.0013(0.0017) 0.45	0.00153(0.0017) 0.36	-0.0034(0.0045) 0.46	-0.0056(0.0041) 0.18
F13	-0.033(0.091) 0.72	-0.049(0.089) 0.58	-0.017(0.38) 0.97	-0.02(0.35) 0.96	0.00017(0.00095) 0.86	0.00012(0.00092) 0.9	-0.0014(0.0044) 0.76	-0.0037(0.004) 0.36
F14	-0.062(0.056) 0.28	-0.048(0.059) 0.42	0.078(0.25) 0.76	0.028(0.24) 0.91	-0.00032(0.00045) 0.48	-0.00023(0.00045) 0.62	-0.000061(0.0028) 0.98	-0.001(0.0026) 0.7
F15	-0.0068(0.0094) 0.47	-0.0094(0.009) 0.31	0.017(0.033) 0.62	0.024(0.03) 0.44	-0.000026(0.000065) 0.69	-0.000046(0.000063) 0.47	0.00014(0.00023) 0.55	0.00014(0.00021) 0.52
F16	-0.0015(0.002) 0.44	-0.0019(0.0019) 0.33	0.003(0.0077) 0.7	0.0037(0.0071) 0.6	-0.000005(0.0000089) 0.58	-0.000007(0.0000085) 0.42	0.00002(0.000033) 0.55	0.000015(0.000031) 0.63
F17	-0.00098(0.0043) 0.82	-0.00068(0.0042) 0.87	0.021(0.012) 0.1	0.022(0.011) 0.071	0.0000025(0.000016) 0.88	0.00000093(0.000015) 0.95	0.000064(0.000036) 0.091	0.000046(0.000034) 0.19
F18	0.0065(0.0049) 0.2	0.0084(0.0048) 0.09	0.02(0.014) 0.18	0.027(0.015) 0.092	0.000019(0.0000093) 0.048*	0.000023(0.000009) 0.016*	0.000036(0.000033) 0.28	0.000021(0.000033) 0.54
F19	0.036(0.023) 0.12	0.047(0.022) 0.047*	0.05(0.058) 0.4	0.039(0.064) 0.55	0.000066(0.000026) 0.014*	0.000079(0.000024) 0.0023*	0.000021(0.000064) 0.75	-0.000052(0.000066) 0.44
F20	0.01(0.11) 0.38	0.13(0.11) 0.25	0.18(0.21) 0.37	0.25(0.24) 0.3	0.000068(0.000081) 0.4	0.000071(0.000079) 0.38	0.000058(0.00015) 0.7	-0.00011(0.00015) 0.48

Relationships between ABI and serum lipid levels were evaluated by univariate or multivariate linear regression analysis adjusted for baseline characteristics. The values are expressed as parameter estimates (standard errors) and *p* values.

* *p*<0.05.

Abbreviations: ABI, ankle-brachial index.

Ln(ABI) was positively associated with lipoprotein particle number in small HDL (F18) and very small HDL (F19) in Stage 4 group (Table 7). In Stage 5 group, although no relationship between ln(ABI) and lipoprotein particle number was observed in univariate regression analysis, ln(ABI) was negatively associated with lipoprotein particle numbers in large VLDL (F5) and medium VLDL (F6).

### CKD-MBD-related markers

Because ln(Klotho) was not associated with baseline characteristics in univariate regression analysis, a multivariate regression model was not examined. In Stage 4 group, univariate regression analysis showed that ln(Klotho) was not associated with cholesterol proportions and lipoprotein particle numbers in lipoprotein fractions (Table 8). In Stage 5 group, ln(Klotho) was negatively associated with the cholesterol proportion in very large HDL (F14). The positive associations between ln(Klotho) and lipoprotein particle number were observed in very small LDLs (F11 and F13), and very small HDLs (F19 and F20).

**Table 8:** Soluble α-Klotho level and cholesterol proportions and lipoprotein particle numbers in lipoprotein fractions.

	Cholesterol proportion		Lipoprotein particle number	
	Stage 4	Stage 5	Stage 4	Stage 5
Fraction	Univariate	Univariate	Univariate	Univariate
F1	-0.023(0.023) 0.32	0.0059(0.036) 0.87		
F2	-0.075(0.066) 0.26	0.053(0.11) 0.65		
F3	-0.051(0.041) 0.22	0.079(0.073) 0.29	-0.025(0.016) 0.12	0.045(0.033) 0.18
F4	-0.029(0.03) 0.33	0.06(0.048) 0.22	-0.0087(0.0068) 0.21	0.019(0.013) 0.14
F5	-0.0062(0.015) 0.68	0.033(0.028) 0.25	-0.0024(0.0028) 0.38	0.0099(0.0051) 0.06
F6	0.0092(0.022) 0.67	0.031(0.038) 0.42	0.00012(0.002) 0.95	0.0065(0.0033) 0.058
F7	0.031(0.046) 0.5	-0.028(0.048) 0.56	0.0013(0.0026) 0.63	0.0028(0.0037) 0.46
F8	0.0042(0.013) 0.74	-0.031(0.016) 0.062	0.000078(0.00058) 0.89	-0.000052(0.001) 0.96
F9	-0.0031(0.014) 0.82	-0.028(0.018) 0.13	-0.00019(0.00033) 0.57	0.00033(0.00051) 0.53
F10	0.0021(0.017) 0.9	0.022(0.022) 0.31	-0.00024(0.00055) 0.67	0.0013(0.00069) 0.067
F11	0.024(0.062) 0.71	0.083(0.064) 0.2	-0.00065(0.0018) 0.71	0.0037(0.0018) 0.048*
F12	0.29(0.25) 0.25	0.16(0.25) 0.52	0.003(0.0049) 0.55	0.0055(0.0057) 0.35
F13	0.26(0.28) 0.35	0.46(0.48) 0.35	0.00013(0.003) 0.96	0.013(0.0056) 0.026*
F14	0.069(0.18) 0.7	-1.02(0.39) 0.013*	0.0002(0.0014) 0.89	-0.007(0.0042) 0.11
F15	0.0037(0.029) 0.9	0.048(0.042) 0.26	-0.000013(0.0002) 0.95	0.00051(0.00026) 0.06
F16	0.000074(0.0062) 0.99	0.0078(0.012) 0.51	-0.0000069(0.000028) 0.8	0.000077(0.000048) 0.12
F17	0.0066(0.013) 0.62	-0.02(0.022) 0.36	-0.0000065(0.000048) 0.89	0.000036(0.000064) 0.58
F18	0.0074(0.016) 0.64	-0.017(0.025) 0.51	-0.0000034(0.000031) 0.91	0.000047(0.000056) 0.41
F19	0.014(0.074) 0.85	0.074(0.1) 0.48	-0.000063(0.000085) 0.47	0.00026(0.000098) 0.012*
F20	0.38(0.32) 0.24	0.29(0.31) 0.36	0.000098(0.00025) 0.69	0.00041(0.00019) 0.036*

Relationships between ln(Klotho) and serum lipid levels were evaluated by univariate linear regression analysis. The values are expressed as parameter estimates (standard errors) and *p* values.

* *p*<0.05.

Abbreviations: ln(Klotho), natural logarithm of serum soluble α-Klotho level.

Univariate regression analysis showed that ln(FGF23) was associated with age [*β* = -0.02207 (0.01151), *p* = 0.059], ln(urinary protein) [*β* = - 0.25102 (0.10031), *p* = 0.015], and ARB use [*β* = 0.5093 (0.269), *p* = 0.063]. In Stage 4 group, ln(FGF23) was not associated with cholesterol proportions and lipoprotein particle numbers in lipoprotein fractions both in univariate and multivariate regression analyses (Table 9). In Stage 5 group, multivariate regression analysis showed that ln(FGF23) was positively associated with cholesterol proportions and lipoprotein particle number in very large HDL (F14).

**Table 9:** FGF23 level and cholesterol proportions and lipoprotein particle numbers in lipoprotein fractions.

	Cholesterol proportion				Lipoprotein particle number			
	Stage 4		Stage 5		Stage 4		Stage 5	
Fraction	Univariate	Multivariate	Univariate	Multivariate	Univariate	Multivariate	Univariate	Multivariate
F1	0.0017(0.07) 0.98	0.0077(0.067) 0.91	0.16(0.12) 0.18	0.21(0.14) 0.14				
F2	-0.029(0.2) 0.88	-0.028(0.19) 0.89	0.46(0.37) 0.22	0.55(0.43) 0.21				
F3	-0.00092288(0.13) 0.99	-0.0036(0.13) 0.98	0.23(0.24) 0.35	0.2(0.26) 0.44	0.003(0.049) 0.95	-0.00015637(0.048) 1	0.085(0.11) 0.45	0.066(0.12) 0.6
F4	0.0066(0.09) 0.94	-0.016(0.089) 0.86	0.14(0.16) 0.38	0.039(0.16) 0.8	0.0017(0.021) 0.94	-0.0027(0.021) 0.9	0.027(0.043) 0.53	0.012(0.041) 0.77
F5	0.043(0.045) 0.34	0.051(0.045) 0.27	-0.013(0.093) 0.89	-0.0051(0.083) 0.95	0.0062(0.0084) 0.46	0.0073(0.009) 0.42	-0.005(0.018) 0.78	-0.0015(0.015) 0.92
F6	0.072(0.064) 0.27	0.039(0.069) 0.57	0.022(0.13) 0.86	-0.099(0.11) 0.38	0.0059(0.0059) 0.33	0.0031(0.0065) 0.63	-0.0094(0.011) 0.42	-0.0077(0.0098) 0.44
F7	0.12(0.14) 0.39	0.085(0.13) 0.52	-0.18(0.15) 0.24	-0.23(0.16) 0.15	0.0072(0.0076) 0.35	0.0067(0.0074) 0.37	-0.019(0.012) 0.12	-0.015(0.012) 0.24
F8	0.0034(0.038) 0.93	0.03(0.037) 0.42	-0.042(0.056) 0.46	-0.024(0.06) 0.7	0.00058(0.0017) 0.74	0.0017(0.0017) 0.32	-0.0037(0.0033) 0.27	-0.0015(0.0038) 0.71
F9	-0.019(0.041) 0.65	0.016(0.041) 0.7	-0.034(0.061) 0.58	-0.0048(0.06) 0.94	-0.00014(0.00098) 0.89	0.00055(0.00096) 0.57	-0.0014(0.0017) 0.42	-0.0000052(0.0017) 1
F10	-0.0017(0.05) 0.97	-0.016(0.048) 0.74	-0.044(0.072) 0.55	-0.018(0.069) 0.8	0.000059(0.0017) 0.97	-0.00018(0.0016) 0.91	-0.0019(0.0024) 0.44	-0.00036(0.0023) 0.87
F11	0.013(0.19) 0.94	-0.035(0.18) 0.85	-0.18(0.21) 0.41	-0.078(0.22) 0.73	0.00064(0.0053) 0.91	0.00031(0.0052) 0.95	-0.0057(0.0063) 0.37	-0.0013(0.0063) 0.84
F12	0.15(0.75) 0.85	-0.3(0.77) 0.69	-0.27(0.83) 0.75	0.15(1.1) 0.89	0.0067(0.015) 0.65	0.00047(0.015) 0.97	-0.0037(0.019) 0.85	0.0072(0.024) 0.77
F13	0.15(0.85) 0.86	0.063(0.85) 0.94	-2(1.6) 0.2	-1.45(1.7) 0.41	0.003(0.0089) 0.74	0.0042(0.0089) 0.64	-0.023(0.019) 0.24	-0.013(0.021) 0.56
F14	0.22(0.54) 0.68	0.45(0.55) 0.42	0.14(1.4) 0.92	2.6(1.2) 0.034*	0.0017(0.0042) 0.68	0.0035(0.0042) 0.41	0.000066(0.014) 1	0.026(0.013) 0.059
F15	-0.0031(0.089) 0.97	-0.039(0.084) 0.65	-0.14(0.14) 0.31	-0.15(0.12) 0.25	0.000094(0.00061) 0.88	-0.00015(0.00059) 0.79	-0.00115(0.00089) 0.2	-0.0012(0.00083) 0.17
F16	-0.0034(0.019) 0.86	-0.008(0.018) 0.65	-0.0091(0.039) 0.82	-0.0054(0.034) 0.88	-0.0000029(0.000083) 0.97	-0.000022(0.000079) 0.78	-0.000076(0.00016) 0.64	-0.00005(0.00015) 0.73
F17	-0.046(0.039) 0.24	-0.054(0.041) 0.19	0.072(0.071) 0.32	0.066(0.065) 0.32	-0.00018(0.00014) 0.2	-0.00017(0.00015) 0.25	0.00015(0.00021) 0.49	0.00015(0.00018) 0.41
F18	-0.0024(0.047) 0.96	-0.00033381(0.046) 0.99	0.12(0.081) 0.16	0.078(0.096) 0.42	0.000016(0.000093) 0.87	0.000035(0.00009) 0.7	0.00019(0.00018) 0.29	0.000095(0.00018) 0.61
F19	0.19(0.22) 0.4	0.13(0.22) 0.57	0.24(0.34) 0.48	0.16(0.35) 0.65	0.00034(0.00025) 0.18	0.00035(0.00026) 0.2	-0.000017(0.00035) 0.96	0.000044(0.00035) 0.9
F20	0.1(0.99) 0.92	-0.45(1) 0.66	0.5(1) 0.63	0.31(1.2) 0.8	0.00028(0.00075) 0.71	0.0002(0.00076) 0.79	-0.00048(0.00064) 0.46	-0.00052(0.00088) 0.56

Relationships between ln(FGF23) and serum lipid levels were evaluated by univariate linear regression analysis. The values are expressed as parameter estimates (standard errors) and *p* values.

* *p*<0.05.

Abbreviations: ln(FGF23), natural logarithm of serum fibroblast growth factor 23 level.

## Discussion

In this study, although no significant difference in the cholesterol levels in lipoproteins was observed between Stage 4 and 5 groups, HPGPC could be used to measure the cholesterol proportions and the particle numbers in the fractions of lipoproteins, which showed significant differences in the lipid profiles between Stage 4 and 5 groups. There has been no report of those subclasses of lipoproteins and lipoprotein particle numbers in CKD patients to the best of our knowledge.

The cholesterol proportions and lipoprotein particle numbers in large VLDL, and small HDL were higher in Stage 4 group than in Stage 5 group. These results are in accordance with previous studies [3, 4]. In CKD patients, decreases in the activities of lipoprotein lipase (LPL) and hepatic lipase lead to the increase in VLDL-C levels [18]. And the decrease in the activity of LCAT and the increase in the activity of cholesterol ester transfer protein (CETP) lead to the decrease in HDL-C level [19–21]. Reductions in the levels of ApoC2 and ApoE, which are donated from HDL to CM and VLDL, decrease LPL activity in CKD patients [22]. The decrease in HDL affects the decrease in CM and VLDL in CKD patients.

In this study, in Stage 4 group, cholesterol proportions and lipoprotein particle numbers in very small HDL were associated with eGFR change rate and rapid eGFR change. There are various studies showing the relationships between dyslipidemia and the loss of kidney function. In the Modification of Diet in Renal Disease (MDRD) study, whose subjects were mainly in Stage 3 or 4, a low HDL-C level was independently associate with a faster loss of kidney function [23]. The Helsinki Heart study in males with dyslipidemia, whose serum creatinine levels were less than 1.3 mg/dl, also showed a negative association between HDL-C level and the loss of kidney function [24]. Some of the pathogenetic factors for CKD progression are local inflammation, endothelial dysfunction, and nitric oxide (NO) deficiency in the kidney [25, 26]. HDL functions as an antioxidant, maintains endothelial function, and is involved in NO production. Dysfunction of HDL aggravates inflammation in local kidney tissue. Moreover, in this study, the lipoprotein particle numbers of large HDL and medium HDL were weakly associated with eGFR change rate in Stage 5 group. This finding suggested that the association between HDL and the loss of kidney function may gradually change with the change in lipid profiles.

Although a low HDL-C level is associated with peripheral arterial disease in the general population [27], in CKD patients, especially in those at late CKD stages, the contribution of HDL-C to peripheral arterial disease has not been established yet. The Multi-Ethnic Study of Atherosclerosis (MESA) showed that, although in persons with eGFR 60 ml/min/1.72m^2^ or higher, the common carotid intima-media thickness (IMT) was associated with HDL-C level, in persons with eGFR lower than 60 ml/min/1.72m^2^, no such association was observed [28]. Cross-sectional studies also showed that HDL-C level was not associated with IMT, or aortic pulse wave velocity in CKD and hemodialysis patients [29, 30]. Our study showed that ABI was positively associated with cholesterol proportions and lipoprotein particle numbers in small HDL and very small HDL in Stage 4 group. The relationship between HDL subclass and ABI in CKD patients has never been reported as far as we know. These lines of evidence suggest that the functional abnormality of HDL more affects atherosclerosis than the quantitative abnormality of HDL-C levels in CKD patients. Oxidized HDLs are dysfunctional HDLs, and their association with atherosclerosis and CVD in CKD patients has been reported [31]. The level of oxidized HDL2 (large HDL) is higher and that of oxidized HDL3 (small HDL) is lower in CKD stage 5 patients than in CKD stage 2 patients [31]. More studies of CKD patients are needed to clarify the role of HDL subclasses and their functions in atherosclerosis.

A longitudinal study showed that subjects with a variant of *KLOTHO* gene (KL-VS) is associated with CVD risk and that the relationship between Klotho and CVD is modified by HDL level [10]. Our study showed that in Stage 5 group, Klotho level was positively associated with the lipoprotein particle number in very small HDLs, and negatively with the cholesterol proportions in very large HDL. A cross-sectional study showed that subjects with homozygous KL-VS had lower HDL-C levels than subjects with homozygous wild-type *KLOTHO* or heterozygous KL-VS [11]. This study also showed that HDL-C level is negatively associated with KL-VS after adjusting for confounders and that HDL-C particle size is associated with KL-VS. From these lines of evidence, Klotho may be associated with HDL-C level and HDL subclasses.

Klotho inhibits signals of insulin/IGF-1 receptors (IR/IGF-Rs), and insulin receptor substrate 1 (IRS1), and induces insulin resistance [32, 33]. In CKD patients, transformation of HDL3 to HDL2 is impaired. HDL2-C level and HDL2-C level/HDL3-C level ratio negatively correlate with insulin resistance [34, 35]. Therefore, Klotho may inhibit HDL transformation through insulin resistance. Moreover, the positive association between Klotho level and lipoprotein particle number in very small LDLs was also observed in this study. *KLOTHO* gene C1818T ploymorphism is associated with LDL-C level in hemodialysis patients and with glucose metabolism in women [36, 37]. Insulin resistance induces small LDL production [38]. Klotho may affect HDL-C and LDL-C levels through insulin resistance.

In this study, although FGF23 level was not found to be associated with HDL-C level, positive relationships of FGF23 level with cholesterol proportion, and lipoprotein particle number in very large HDL in Stage 5 group were observed. A cross-sectional study showed that FGF23 level is not associated with HDL-C level in 80 nondialysis CKD patients [39]. A cohort study showed that FGF23 level is negatively associated with HDL-C levels in 654 hemodialysis patients [40]. Another cohort study showed that FGF23 level is not associated with HDL-C level, but negatively associated with non-HDL-C level in 196 hemodialysis patients [41]. These inconsistent results may be due to differences in statistical power and lipid measurement methods used [40]. And the relationship between FGF23 and HDL may change depending on CKD stage. However, these studies suggest that FGF23 is associated with lipid metabolism. A candidate hypothesis proposed in previous reports is that FGF23 can signal through receptors of other FGF families such as FGF21, which regulates lipolysis [40–42]. An observational study showed an independent relationship between FGF23 level and insulin sensitivity [43]. FGF23 may affect HDL metabolism though insulin sensitivity. Klotho functions as a cofactor of FGF23 and mediates the effects of FGF23. However, there has been no report about the combined effects of Klotho and FGF23 on lipid metabolism. Large-scale studies should be carried out to establish the relationship between FGF23, Klotho, and lipid metabolism.

HDL is expected to be a target of therapy against dyslipidemia. However, intensive clinical trials of CETP inhibitors such as torcetrapib and dalcetraib did not show clinical benefits [44, 45]. Niacin could increase HDL levels, but could not inhibit coronary artery disease [46]. It has been reported that cholesterol efflux capacity of HDL has a strong inverse association with coronary artery disease independently of the HDL cholesterol level in patients with coronary artery disease [47]. Uremia impairs HDL function in hemodialysis patients [22]. There is a necessity for therapies based on not only a simple increase in HDL-C level but also improvement of HDL functions including cholesterol efflux [48]. Because there are various subclasses in HDL with different functions, the profile of HDL subclasses in patients after a therapy against dyslipidemia may not be the same as that in healthy people. Measurement of HDL subclasses is useful as a monitor of HDL therapies.

HDL subclass (HDL2 and HDL3) is associated with coronary heart disease. However, the results are not consistent because of the measurement methods. Studies of the measurement of HDL2 by gradient gel electrophoresis showed consistent results [49]. On the other hand, the results of the studies of the measurement of cholesterol levels of HDL2 and HDL3 were inconsistent. Therefore, we measured both cholesterol proportions and lipoprotein particle numbers in lipoprotein fractions.

This study has several limitations. First, this study involved 71 patients at CKD stages 4 and 5, which may be insufficient for determining the relationship between lipoprotein fraction, the loss of kidney function, atherosclerosis, and CKD-MBD. Moreover, since the study period was 6 months, we were unable to examine the longitudinal changes in laboratory findings over time. Long-term observational studies including early CKD will provide us more information about the relationship. Second, the effects of insulin resistance and antihyperlipidemic drugs on dyslipidemia were not investigated. Third, as a marker of atherosclerosis, only ABI was measured. Measurements of various markers such as IMT and interleukins will enable us to evaluate the relationships between lipid profiles and atherosclerosis in terms of loss of kidney function. Fourth, because this was an observational study, the effect of lipid profiles on the loss of kidney function has remained unclarified. Interventional studies are needed to clarify the causative role of lipoprotein fractions in the loss of kidney function. Fifth, although there are various risk factors of CVD, we could investigate only two CKD-MBD related factors, Klotho and FGF23.

## Conclusions

This study showed the difference in lipid profiles between CKD stages 4 and 5, and that different HDL subclasses are associated with the loss of kidney function, ABI, and Klotho and FGF23 levels depending on the CKD stages. More longitudinal and interventional studies are needed to clarify the different roles of subclasses in HDL in CKD progression and atherosclerosis in CKD patients.
